# Iron Deficiency Prevention, Screening, and Treatment: A Quality Improvement Initiative Introducing Reticulocyte Hemoglobin in a Level III Neonatal Intensive Care Unit

**DOI:** 10.3390/nu17213391

**Published:** 2025-10-29

**Authors:** Narmin Javadova, Pamela J. Kling, Sally Norlin, Whitley N. Hulse

**Affiliations:** 1 Department of Pediatrics, Division of Neonatology, University of Wisconsin, Madison, WI 53792, USA; njavadova@uwhealth.org (N.J.); pkling@wisc.edu (P.J.K.); sally.norlin@unitypoint.org (S.N.); 2 Neonatology, UnityPoint Health—Meriter Hospital, 202 S. Park St., Madison, WI 53715, USA

**Keywords:** iron deficiency, preterm neonates, small-for-gestational age, reticulocyte hemoglobin

## Abstract

**Objective**: To implement a neonatal iron deficiency (ID) guideline as part of a neuroprotective strategy using reticulocyte hemoglobin content (RET-He) for neonates born <33 weeks postmenstrual age (PMA) and small for gestational age (SGA) neonates ≥33 weeks PMA, to achieve ≥80% screening rate by June 2024. **Methods**: An interdisciplinary team conducted a quality improvement initiative in a level III neonatal intensive care unit (NICU) from April 2022 to August 2024. RET-He is a validated, sensitive marker of early iron deficiency reflecting recent iron supply for erythropoiesis and providing a more reliable measure than ferritin. The primary outcome was RET-He screening at 30 ± 7 days for neonates <33 weeks PMA or pre-discharge for SGA neonates ≥33 weeks PMA. Exclusion criteria were death or transfer before eligibility. Process measures included ID screening failure rate (RET-He level < 29 pg). **Results**: Of 345 eligible neonates, P-chart analysis showed screening rates for premature neonates <33 weeks PMA declined during PDSA 1–2, before improving to 85.9% in PDSA 3. ID screening failure was 12.6% at one month, increasing to 32.1% at two months. For SGA neonates ≥33 weeks PMA, screening rates remained low, peaking at 36% in PDSA 3, with a 2.2% failure rate. **Conclusions**: Implementation of a RET-He based ID guideline improved screening rates for premature neonates but was less effective for SGA neonates. Despite improved guideline adherence, ID prevalence remained high at NICU discharge, indicating a further need to improve nutritional prevention and treatment strategies.

## 1. Introduction

Iron deficiency (ID) is the most common micronutrient deficiency worldwide [1,2,3,4,5]. The body prioritizes iron for erythropoiesis over other organs, including the brain [6]. Thus, neonatal ID is associated with potentially irreversible impairments in motor skills, cognitive function, social-emotional development, and neurophysiological growth that persist even after iron repletion [1,7,8,9,10]. Neonatal ID may result from limited placental iron transfer due to maternal ID, maternal hypertension or pre-eclampsia, intrauterine growth restriction, small for gestational age (SGA) status, or prematurity. Increased fetal iron demand, as in maternal diabetes, also contributes to neonatal ID [1,2,10]. Premature infants face additional postnatal risk factors for ID, including rapid growth velocity, immediate cord clamping, delayed enteral iron administration, and frequent phlebotomy [11,12]. Early prevention, identification, and treatment are therefore critical, and ID screening may be considered part of a broader neuroprotective strategy in at risk neonates, including those that are born premature or SGA [6].

The American Academy of Pediatrics (AAP) recommends iron supplementation for premature infants at 2–4 mg/kg/day beginning at 2–4 weeks of life, with screening for ID between 9–12 months of corrected age [6,13,14]. Yet, 20–30% of NICU infants are diagnosed with ID before discharge, highlighting the need for improving nutritional prevention strategies and early screening [3,15].

Serum ferritin, a common ID biomarker, has several limitations in neonates: it is affected by inflammation, requires additional blood sampling, and is not well validated in infancy—especially in extremely premature infants. These limitations make ferritin measurements difficult to interpret, and its use in evaluating iron deficiency in neonates is insufficient [4,8,10].

Reticulocyte hemoglobin content (RET-He) has emerged as a validated, dynamic, and sensitive biomarker for pre-anemic ID, reflecting iron availability for erythropoiesis over the preceding seven days [11,16,17,18]. Unlike ferritin, RET-He is unaffected by inflammation, can be measured from the same blood sample used for a CBC, responds rapidly to changes in iron incorporation, improving diagnostic accuracy in the NICU [3,11,19]. Animal studies show that RET-He is the only CBC biomarker to decline before the onset of brain ID, supporting its value for early detection [5,7,20].

A review of our baseline iron screening quality improvement data at the University of Wisconsin and UnityPoint Health—Meriter Hospital (June 2016 to December 2018) revealed 80% compliance with ID screening at one month of age, with 16% diagnosed with ID using a ferritin threshold of <70 µg/L for the combined cohort [21,22]. Following the introduction of RET-He in the clinical laboratory, we implemented a standardized neonatal ID prevention, screening, and treatment guideline as part of a neuroprotective strategy, using the Model of Improvement framework [23,24]. Our initiative aimed to screen neonates born <33 weeks postmenstrual age (PMA) at 30 ± 7 days of life and SGA neonates born ≥33 weeks PMA before discharge, using RET-He, targeting an 80% screening rate by June 2024.

## 2. Methods

### 2.1. Context

Our NICU is a 42-bed Level III NICU in a tertiary-care medical center, with approximately 600 annual admissions. The NICU admits neonates born <35 weeks PMA at birth, those with birthweight < 2000 g, neonates with complex congenital anomalies, neonates requiring mechanical ventilation, inhaled nitric oxide, therapeutic hypothermia, or neonates who fail the postnatal transition to remain in the mother’s room. Placental transfusion with delayed umbilical cord clamping at birth was standardly performed for all neonates, unless a contraindication, such as abruption or cord compromise. Unit feeding protocol was in place with either maternal or donor milk. Once enteral feeding tolerance of ≥60 mL/kg/day is established, iron supplementation is initiated at 2 weeks of age with 2–3 mg/kg of ferrous sulfate (15 mg of elemental iron/mL), which is in alignment with the AAP recommendations to initiate iron supplementation by 2–6 weeks of life [14]. RET-He measurements became available in March 2022 on the Sysmex hematologic analyzer (Model XN-10, Sysmex America, Inc., Lincolnshire, IL, USA), which provides concordant levels to the Bayer ADVIA 2120 analyzer that measures reticulocyte hemoglobin content (CHr) [25]. All measurements were performed following Unity-Point/Meriter Laboratory Services’ standard operating procedures and the manufacturer’s instructions. Using the Quality Improvement Screening Tool of UnityPoint Health—Meriter and the University of Wisconsin-Madison, this project was deemed a quality improvement initiative. Data management practices were reviewed and approved by the University of Wisconsin-Madison and complied with institutional and HIPAA standards of data confidentiality. The UnityPoint Health—Meriter clinical laboratory provided all RET-He values obtained in the NICU, along with the corresponding names and medical record numbers. Institutional approval was also granted to link site-specific, de-identified Vermont Oxford Network (VON) data via a secure key to provide demographic data on these neonates.

### 2.2. Inclusion and Exclusion Criteria

Eligible neonates included those born <33 weeks PMA or were diagnosed as SGA (≤10th percentile on Fenton growth curve) at ≥33 weeks PMA [26]. Neonates were excluded if they died or transferred to another facility before recommended screening at 1 month of age for premature infants (<33 weeks PMA) or before NICU discharge for SGA infants. Their data was not analyzed.

### 2.3. Intervention

**Figure 1:**
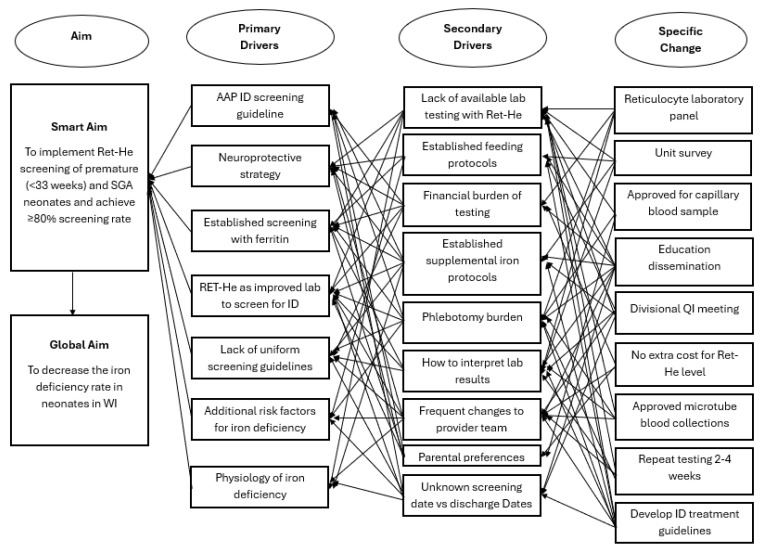
Key Driver Diagram that shows aim, primary drivers, secondary drivers, and specific changes. Ret-He: reticulocyte hemoglobin, ID: iron deficiency, AAP: American Academy of Pediatrics, WI: Wisconsin, SGA: Small for Gestational Age, QI: quality improvement.

A new interdisciplinary quality improvement team in the NICU was formed in January 2022, focusing on ID prevention, screening utilizing RET-He, and treatment. Key stakeholders included two neonatal intensivists, a pediatric resident, a neonatal dietitian, a laboratory information systems analyst, and a neonatal pharmacist. A key driver diagram (Figure 1) outlines changes to practice, incorporating specific changes and primary and secondary drivers identified through a unit-wide survey. A prospective quality improvement project was designed with nine specific changes that were organized into three Plan-Do-Study-Act (PDSA) cycles, each with individual sub-aims. Unit learning from PDSA 1 and 2 was applied in the design of PDSA 3. The first two PDSA cycles focus on appropriate screening with RET-He, and the third PDSA cycle summarizes prevention, screening, and treatment into a comprehensive guideline. Data was analyzed quarterly to track changes through individual PDSA cycles, which influenced the interventions and timing of introducing subsequent PDSA cycles.

### 2.4. Measures

As part of a neuroprotective strategy for neonates, our primary outcome measure was measuring RET-He for ID screening at 30 ± 7 days for infants born <33 weeks PMA or pre-discharge for SGA neonates born ≥33 weeks PMA. The timing of ID screening at one month for preterm infants was determined to minimize painful pokes by coordinating blood draw with other established 1-month nutritional labs and guide therapy as neonates entered their physiologic hematopoietic nadir that occurs between 4–8 weeks for preterm neonates [27]. Capillary samples were utilized unless a neonate had a venous or arterial line that could be used for sampling. Process measures included ID screening failure rate, defined as RET-He < 29 pg [4,11,18]. Karagülle et al. established that RET-He level < 29 pg provided 90.6% sensitivity and 66.7% specificity in the diagnosis of ID [4]. As the initiative began, feedback was provided on how to evaluate resolution or sustainment of ID following a low RET-He measurement. Two process measures were added in response: percentage of neonates with an appropriate increase in iron supplementation in response to a low RET-He measurement and percentage of neonates with repeat RET-He screening at 2 months of life, if infants remained hospitalized. Balancing measures included retrospective collection of the percentage of neonates diagnosed with any Bell’s stage of necrotizing enterocolitis (NEC) or late-onset (enteral iron correlated with Gram-negative sepsis that are siderophilic) to ensure that increased enteral iron supplementation did not alter risk factors [28,29,30].

**PDSA 1**: The aim was to implement RET-He as the screening lab for ID at 1 month of life for infants born <33 weeks PMA or who were diagnosed as SGA and born ≥33 weeks PMA before discharge. We utilized Pediatric Grand Rounds in February 2022 to introduce RET-He as the ID screening measurement, educating physicians, advanced practice providers, and pediatric residents on the targeted population and how to interpret RET-He measurement results. We coordinated with the laboratory information services through UnityPoint Health—Meriter Hospital and its electronic medical record to develop a reticulocyte panel order that was approved in March 2022. This panel consists of five parameters: red blood cell count, reticulocyte percentage, absolute reticulocyte count, immature reticulocyte fraction, and RET-He. RET-He can be collected in an EDTA microtube used for CBC with a targeted blood volume of 0.25–0.5 mL per sample. ID screening with RET-He went live in April 2022. All RET-He tests collected in the NICU were captured in the laboratory information systems report.

**PDSA 2**: The aim was to add a second ID screening at 2 months of life for infants born <33 weeks PMA or for those born ≥33 weeks PMA with SGA who remained admitted in the NICU. During the review of the first two quarters of data, a significant rise in the diagnosis of ID (RET-He < 29) was noted among premature infants who also underwent an additional screening at 2 months of age. Education on this additional testing, while reinforcing the initial 1-month screening, was provided during didactic education for pediatric residents in August 2022 and at a quality improvement and guidelines meeting for the Division of Neonatology in September 2022.

**Figure 2:**
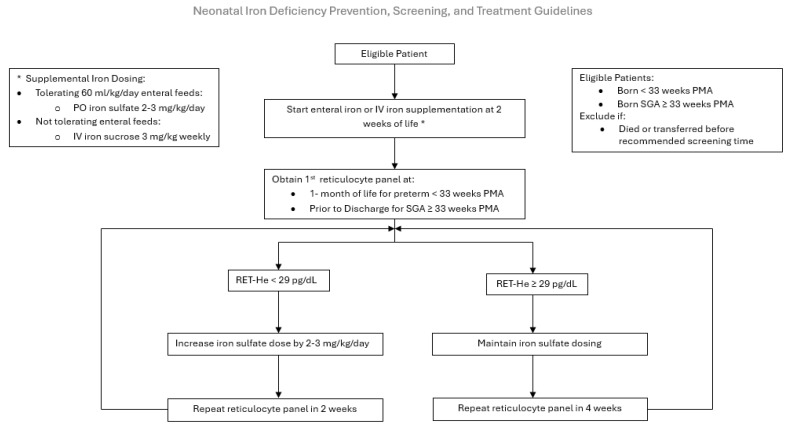
Neonatal Iron Deficiency Prevention, Screening, and Treatment Guideline. PO: per oral, IV: intravenous, PMA: postmenstrual age, Ret-He: Reticulocyte hemoglobin.

**PDSA 3**: The aim was to improve screening rates by disseminating the comprehensive Neonatal ID Prevention, Screening, and Treatment Guideline in May of 2023 (Figure 2). Our NICU feeding protocol relies on the use of maternal or donor human milk, fortified with human milk fortifiers, for all premature infants. Human milk is low in iron and provides a mean of 1 mg/kg/day of supplemental iron, insufficient to support premature infant growth. Iron supplementation is initiated at 2 weeks of age. The route, enteral versus parenteral, is determined by the neonate’s enteral feeding tolerance. If tolerating ≥ 60 mL/kg/day of enteral feeds, 2–3 mg/kg of oral ferrous sulfate (15 mg of elemental iron/mL) is scheduled. Our pharmacy provides a unit dose of 3 mg of elemental iron per dose of ferrous sulfate. The guideline increases the frequency of dosing based on the infant’s weight to achieve a dose of 2–3 mg/kg/day, as recommended by the AAP [6,13,14]. For example, an infant weighing 1 kg would receive once daily dosing. If NPO or not tolerating sufficient enteral feeds at 2 weeks of life, a 3 mg/kg once weekly infusion of IV iron sucrose is initiated until adequate enteral feeds are established to transition to oral iron supplementation. If the RET-He measurement is ≤29 pg, the infant is diagnosed with ID, and their iron supplementation is increased by 2–3 mg/kg/day. Repeat RET-He testing is performed 2 weeks later if the infant is iron-deficient or 4 weeks later (at 2 months of life) if the infant is iron-sufficient. This cycle continues until the infant is discharged home with a recommendation to continue iron supplementation until one year corrected age. Iron supplementation is not discontinued if the infant receives a red blood cell transfusion, and erythrocyte-stimulating agents are not routinely utilized in our NICU.

### 2.5. Studying the Intervention

Eligible RET-He levels in neonates were identified through a manual review of the RET-He laboratory data collected on NICU patients, which were provided securely from the UnityPoint—Meriter hematology laboratory every 3 months (quarterly). Deidentified demographic and outcome data from eligible neonates were then obtained from UnityPoint Health—Meriter site’s Vermont Oxford Network (VON) data and connected to available RET-He levels in neonates through a secure key. Data was confirmed by manual review, with any missing data being manually collected. Baseline data were obtained from a previous study that utilized ferritin as a screening measurement for 257 infants from June 2016 to December 2018. Prospective implementation data were collected and reviewed quarterly, based on a mean of 20–30 eligible patients per quarter.

### 2.6. Analysis

Demographic data, including mean values, were analyzed using a Z-test to assess the difference between groups for both continuous and categorical variables; no multiple comparison was completed. Statistical analysis was performed with Excel (version 1808, Microsoft, Redmond, WA, USA). Statistical process control charts for the primary outcome, process, and balancing measures were created with QI Macros for Excel (KnoWare International, Denver, CO, USA) [31]. P-charts were utilized for primary outcome measures to monitor proportion, trends, and process stability [32]. Special cause change was evaluated using the Institute for Healthcare Improvement rules [24]. Upon entering the sustainment phase, the center line was locked to monitor the system’s performance following active intervention.

## 3. Results

### 3.1. Demographics

Baseline data included 257 eligible infants from June 2016 to December 2018. Our current quality improvement project involved 345 eligible infants. The intervention phase ran from May 2022 to December 2023, during which data were collected from 263 infants across the three PDSA cycles. The sustainment phase, which ran from January 2024 to August 2024, involved collecting data from 82 infants. Compared with baseline demographic data, the implementation/sustainment groups had higher proportions of minority mothers, lower birth-weight z-scores, and SGA infants ≥ 33 weeks PMA, but fewer premature infants <33 weeks PMA (Table 1).

### 3.2. Primary Outcomes:

**Figure 3:**
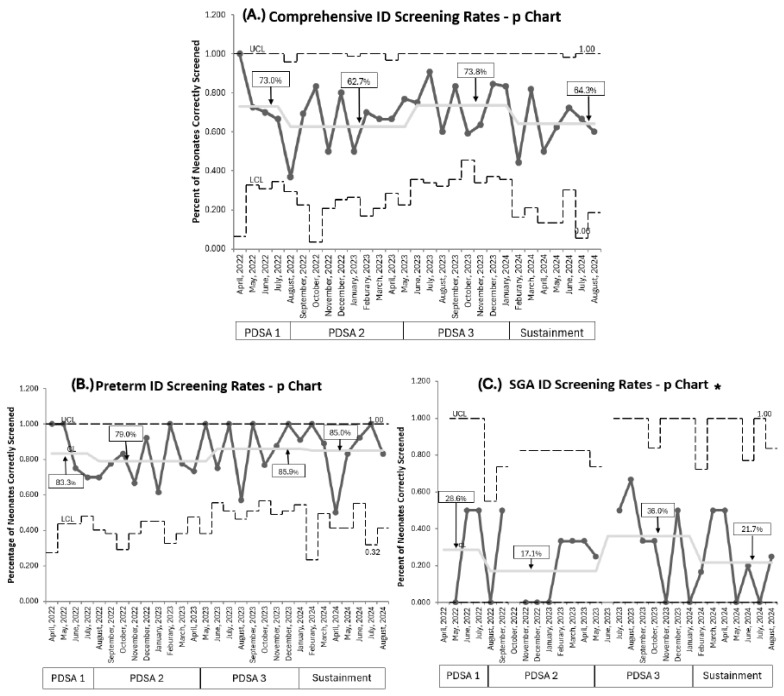
P Charts. (**A**). Comprehensive combined population ID screening rates. (**B**). Premature neonates <33 weeks PMA ID screening rates. (**C**). SGA neonates ≥33 weeks PMA ID screening rates. PDSA cycles are noted at the bottom of each graph. Centerline adjusted for each new PDSA and sustainment cycle, with a percentage for each cycle in square; no special cause variation noted per IHI rules. ID: iron deficiency, PMA: postmenstrual age, SGA: small for gestational age, UCL: upper control limit, LCL: lower control limit. * The UCL line is discontinuous if no neonates were eligible that month for ID screening.

Baseline screening compliance with ferritin was 80% (257/323) [21]. Current overall comprehensive RET-He screening compliance, combining both populations throughout all PDSA cycles, was 67.2% (232/345). RET-He screening was lower (73%) during PDSA 1. Compliance in PDSA 2, despite educational efforts to address knowledge gaps, remained below target (62.3%) with a wider variation per month, demonstrated by a wider confidence interval. Implementing PDSA 3 improved screening to 73.8%, which approached our goal; however, it declined in the sustainment phase to 64.3%. No special cause variation was detected throughout (Figure 3A).

Comparing our two target populations, RET-He screening rates were higher for premature infants born <33 weeks PMA than in SGA infants born ≥33 weeks PMA. For premature infants born <33 weeks PMA, screening rates eventually achieved 85.9% during PDSA 3 and were maintained at 85% throughout the sustainment period (Figure 3B). Overall, 209 of 254 (82.2%) premature infants were accurately screened at 1 month of age. On *post hoc* analysis, we identified that premature infants born at 32 weeks PMA accounted for 82% (37/45) of premature infants whose ID screening was missed. This subgroup of premature infants had a mean NICU length of stay of 27 days. For SGA infants ≥33 weeks PMA, screening rates remained low despite recurrent PDSA cycles, peaking at 36% in PDSA 3 before falling to 21.7% during sustainment (Figure 3C). The mean NICU length of stay for SGA neonates was 18 days.

### 3.3. Process Measures

Baseline ID prevalence at 1 month (ferritin < 70 µg/L) was 16% [21]. ID prevalence using RET-He was 12.6% (32/254) at 1 month of life. Nearly all neonates (93.8%, 30/32) diagnosed with ID received appropriate supplemental iron dosing increase by 2–3 mg/kg/day. Following PDSA cycle 2, 81.6% (84/103) of premature neonates who remained admitted underwent repeat screening, with ID prevalence rising to 32.1% (27/84).

Only 2.2% (2/91) of SGA neonates born at ≥33 weeks PMA were diagnosed with ID before discharge; both were given an appropriate increase in their supplemental iron dosing post diagnosis. Only 1 of the 91 SGA neonates remained admitted at 2 months of life, had the anticipated repeat RET-He testing, and were iron-sufficient.

### 3.4. Balancing Measures

Balancing measures remained stable throughout our study. Rates of NEC remained low at 0.5% (2/345 comprehensive; 2/254 premature; 0/91 SGA), below the national mean of 2–5% [29]. Late-onset sepsis occurred in 7.8% (27/345 comprehensive; 27/254 premature; 0/91 SGA) of all neonates, consistent with national rates of 8–9% [28].

## 4. Discussion

### 4.1. Primary Outcome

Our quality improvement initiative successfully implemented standardized ID prevention, screening, and treatment guidelines using RET-He as part of a neuroprotective strategy for neonates born <33 weeks PMA at 1 month of life, achieving a screening compliance of 85%. We did not achieve our primary aim of pre-discharge screening SGA neonatal cohort born ≥33 weeks PMA, as we only achieved a compliance rate of 36%, which declined to 21.7% in sustainment. The greater-than-expected decline in sustainment of comprehensive screening likely reflects the impact of staffing transitions and evolving institutional processes, which highlights the need for strategies to support long-term practice adherence. Despite strong adherence to supplemental treatment guidelines, ID prevalence in premature neonates remained high throughout the first month of life. ID prevalence nearly doubled, rising from 12.6% to 32.1%, by 2 months of life using the same RET-He of <29 pg despite an adequate increase in supplemental iron dosing, suggesting the current prevention strategies may be insufficient to meet the iron demands of premature neonates fully.

### 4.2. Clinical Implications

The body preferentially delivers available iron into red blood cells, with anemia a late manifestation of ID and likely after the brain has experienced iron depletion during critical times of neurodevelopment [1,7,33]. This highlights the importance of early pre-anemic iron screening to detect ID as soon as possible. Measurable blood iron biomarkers (ferritin, transferrin saturation, serum iron/transferrin saturation, soluble transferrin receptor, mean corpuscular volume) do not accurately reflect the brain’s current iron status [5,23]. RET-He confers the advantage that it is the only CBC biomarker that declines before the onset of brain ID in the nonhuman primate model [1,5,20].

Despite high adherence to the AAP recommendations for early iron supplementation and consistent laboratory monitoring, we observed high rates of ID at one month of life, with prevalence increasing at 2 months of life. These results highlight how difficult it remains to prevent ID in these vulnerable groups and calls for improved nutritional strategies and more efficient ways to screen, diagnose, and treat ID to reduce long-term neurodevelopmental risk. The AAP recommends a minimum of 2–4 mg/kg/day of supplemental iron from one through twelve months of life for premature or high-risk neonates, recognizing that breast milk is inherently low in iron and that older infants often consume inadequate amounts of iron-rich solid foods. Our institutional guidelines are consistent with this recommendation, which involves recommending placental transfusion and initiating iron supplementation at two weeks of life and maintaining it throughout their hospitalization, regardless of whether they receive a postnatal red blood cell transfusion. However, our findings suggest that our current dosing strategy may be inadequate to meet the increased daily iron requirements of premature neonates, particularly during the period of rapid postnatal weight growth beginning between one and two months of life.

This may be further influenced by limited understanding of factors affecting enteral iron absorption, such as inflammation-induced hepcidin regulation, dosing frequency and timing of supplemental iron, and red blood cell transfusions [1,2,11,34]. The RET-He diagnostic threshold of 29 pg at 1 month may not be indicative of ID at all time periods, suggesting that perhaps a different or higher threshold for RET-He at 1 month of life can predict the development of ID at 2 months of life, when the rates doubled. This is reflected in the reference intervals established by Christensen et al., which showed higher RET-He levels at 30 days of life compared to 60 days of life [35].

### 4.3. High- Risk Populations

Two particularly vulnerable populations emerged from our analysis. First, premature neonates born at 32 weeks PMA, who were due for ID screening near NICU discharge. Their mean length of stay was 27 days. Competing priorities in preparation for discharge may have contributed to missed evaluations, leaving them at risk for unrecognized ID despite multiple risk factors. Similarly, SGA neonates born ≥33 weeks PMA experience persistently low screening rates, who also had relatively short NICU stays, which limited opportunities for screening. We had a relatively small sample size of SGA neonates ≥33 weeks PMA, which may have limited the success of screening implementation and reduced the reliability of statistical interpretation. Furthermore, the low prevalence of ID at the time of discharge in SGA neonates born ≥33 weeks PMA raises uncertainty about whether inpatient screening accurately identifies those most likely to develop ID later accompanying rapid post-discharge growth typical of SGA neonates. Future research into alternative outpatient testing with RET-He at 1–2 months of age may identify those at high risk for developing ID that are not represented by testing SGA neonates during their inpatient NICU course. Or it may identify other inpatient approaches, with other iron blood biomarkers, that better predict future risk of developing ID [36,37]. Both the cohorts born at 32 weeks PMA and SGA >33 weeks PMA remain at high risk for ID and adverse neurobehavioral outcomes and would benefit from targeted interventions to improve their screening rates.

## 5. Limitations

Our quality improvement study has several limitations. As a single-center initiative with a relatively small sample of SGA neonates, the generalizability of our findings may be limited. We applied a uniform RET-He threshold of <29 pg to define ID across all time points, which enhanced guideline clarity and consistency. However, the optimal cutoff may differ and warrants further investigation. Additionally, the relatively short NICU course for some patients may have constrained the intervention’s impact, and missed screenings could have influenced ID prevalence calculations. ID is known to influence long-term neurodevelopmental outcomes; however, assessment of these outcomes was beyond the scope of this project. Despite these limitations, our study provided important preliminary insights and a practical framework for implementing neonatal ID screening, prevention, and treatment guidelines.

## 6. Conclusions

A comprehensive Neonatal ID Prevention, Screening, and Treatment Guideline, using RET-He, was successfully implemented for premature neonates as part of a neuroprotective strategy. Screening rates were sustained above 85% for neonates < 33 weeks PMA. However, neonates born at 32 weeks PMA and SGA neonates born ≥33 weeks PMA remain high-risk groups with low screening adherence. These groups require additional targeted interventions, such as adding iron screening to the discharge checklist or automated reminders. Additional clinical research is needed to determine optimal initial supplemental iron nutritional strategies, as current practices remain insufficient to prevent ID in approximately one-third of premature neonates.

## Figures and Tables

**Table 1:** Demographics for baseline and comprehensive quality improvement (QI) initiative, including intervention and sustainment phases. A. Maternal Factors. B. Fetal Factors. PMA: postmenstrual age.

**A. Maternal Factors**	**Baseline Demographics** **N = 257**	**QI Initiative Comprehensive Demographics** **N = 345**	***p* Value**
Maternal Minority	59 (23.1%)	108 (31.3)	*p* = 0.02
Maternal Diabetes	36 (13.9%)	55 (15.9%)	0.51
Hypertension/Preeclampsia	86 (33.3%)	138 (40%)	0.1
**B. Fetal Factors**	**Baseline Demographics** **N = 257**	**QI Initiative comprehensive demographics** **N = 345**	***p* value**
Premature < 33 weeks PMA	227 (88.3%)	254 (73.6%)	*p* < 0.01
Gestational Age at Birth	31.5 ± 2	31.5 ± 3.8	1.0
Birthweight		1501 g ± 515	
Birth Weight *Z*-Score (mean)	−0.21	−0.6	*p* < 0.01
Sex Male %	136 (53.1%)	185 (53.6%)	0.87
Small for Gestation ≥ 33 weeks PMA	30 (11.7%)	91 (26.4%)	*p* < 0.01
Multifetal Gestation	75 (29.2%)	111 (32.2%)	0.43

## Data Availability

The original contributions presented in this study are included in the article. Further inquiries can be directed to the corresponding author.
